# Solution Processed PVB/Mica Flake Coatings for the Encapsulation of Organic Solar Cells

**DOI:** 10.3390/ma14102496

**Published:** 2021-05-12

**Authors:** Iftikhar Ahmed Channa, Ali Dad Chandio, Muhammad Rizwan, Aqeel Ahmed Shah, Jahanzeb Bhatti, Abdul Karim Shah, Fayaz Hussain, Muhammad Ali Shar, Abdulaziz AlHazaa

**Affiliations:** 1 Department of Materials and Metallurgical Engineering, Faculty of Chemical and Process Engineering, NED University of Engineering and Technology, University Road, Karachi 75270, Pakistan; alidad@neduet.edu.pk (A.D.C.); engr.rizwan@neduet.edu.pk (M.R.); aqeelshah@neduet.edu.pk (A.A.S.); jahanzebbhatti@neduet.edu.pk (J.B.); 2 Department of Mechanical Engineering, Faculty of Engineering, University of Malaya, Kuala Lumpur 50603, Malaysia; 3 Department of Chemical Engineering, Dawood University of Engineering and Technology, Karachi 74800, Pakistan; abdulkarim@duet.edu.pk; 4 Modeling Evolutionary Algorithms Simulation and Artificial Intelligence, Faculty of Electrical & Electronics Engineering, Ton Duc Thang University, Ho Chi Minh City 700000, Vietnam; fayaz@tdtu.edu.vn; 5 King Abdullah Institute for Nanotechnology, King Saud University, Riyadh 11451, Saudi Arabia; mashar@ksu.edu.sa (M.A.S.); aalhazaa@ksu.edu.sa (A.A.); 6 Department of Mechanical & Energy Systems, Faculty of Engineering and Informatics, University of Bradford, Bradford BD7 1DP, UK; 7 Research Chair for Tribology, Surfaces and Interface Sciences, Department of Physics and Astronomy, College of Science, King Saud University, Riyadh 11451, Saudi Arabia

**Keywords:** thin barrier films, synthetic mica composite, flexibility, transparency, solar cell protection

## Abstract

Organic photovoltaics (OPVs) die due to their interactions with environmental gases, i.e., moisture and oxygen, the latter being the most dangerous, especially under illumination, due to the fact that most of the active layers used in OPVs are extremely sensitive to oxygen. In this work we demonstrate solution-based effective barrier coatings based on composite of poly(vinyl butyral) (PVB) and mica flakes for the protection of poly (3-hexylthiophene) (P3HT)-based organic solar cells (OSCs) against photobleaching under illumination conditions. In the first step we developed a protective layer with cost effective and environmentally friendly methods and optimized its properties in terms of transparency, barrier improvement factor, and bendability. The developed protective layer maintained a high transparency in the visible region and improved oxygen and moisture barrier quality by the factor of ~7. The resultant protective layers showed ultra-flexibility, as no significant degradation in protective characteristics were observed after 10 K bending cycles. In the second step, a PVB/mica composite layer was applied on top of the P3HT film and subjected to photo-degradation. The P3HT films coated with PVB/mica composite showed improved stability under constant light irradiation and exhibited a loss of <20% of the initial optical density over the period of 150 h. Finally, optimized barrier layers were used as encapsulation for organic solar cell (OSC) devices. The lifetime results confirmed that the stability of the OSCs was extended from few hours to over 240 h in a sun test (65 °C, ambient RH%) which corresponds to an enhanced lifetime by a factor of 9 compared to devices encapsulated with pristine PVB.

## 1. Introduction

Most of the active materials used in organic solar cells degrade due to reactions of oxygen and moisture [1,2,3]. To reduce this degradation, the process of encapsulation is carried out for the protection of devices [4]. The quality of the barrier materials determines the life of organic electronic devices [5,6]. Hauch et al. demonstrated that an encapsulatingbarrier film having a water vapor transmission rate (WVTR) of 10^−3^ g⋅m^−2^⋅day ^−1^@ 25 °C/40% RH may extend the life of organic solar cells to beyond 3 years [6]. As a common practice, encapsulating films are laminated from both sides of the solar cells and a lamination is used between flexible protective films [7,8]. It provides a device with a lifetime of several years under favorable circumstances, i.e., 25 °C/85% RH [7,9].

Thus, developing alternative approaches of protecting printed devices is a demanding trend. Coating of protective layers on top of devices using solutions is one of most employed strategies. At present, coated barrier layers are far from the quality of encapsulated barriers, but, in some cases, they can be utilized effectively. For instance, a short-term protection of devices between production and final encapsulation. Therefore, barrier films exhibiting oxygen and moisture permeabilities of ~0.12 cm⋅cm^3^⋅m^−2^ ⋅day^−1^⋅bar^−1^ and ~0.05 cm⋅g⋅m^−2^⋅day^−1^, respectively, can be used for the temporary protection of solar cell devices [4]. Additionally, encapsulation as a protective layer should not disturb the basic characteristics of devices. Hence, a protective material must be flexible, lightweight, optically transparent, and economical [10,11,12].

Lots of materials are being used and have been demonstrated for solar cell encapsulation, including metals, polymers, glass, and ceramics. Among all these options, polymers are presently widely used due to their inherent low density, cost effectiveness, as well as the diversity of their physiochemical stabilities [13,14,15]. One of the main advantages of polymers is their easy processability, since they can be processed using simple coating techniques, such as spin coating, doctor blading, spray coating, etc. In the food packaging industry, 40% of the market share is being performed by polymers [16]. Except for polymers, all other materials are extremely rigid but offer high-quality barrier characteristics. The barrier characteristics against any specific permeating molecule can be enhanced by various factors, for instance, ethylene vinyl alcohol (EVOH) offers outstanding barrier characteristics to oxygen in a dry environment. However, in a moist environment (e.g., >75% relative humidity), its rate of oxygen transmission can be increased by one order of magnitude, owing to the swelling of the polymer in a water environment [17,18].

In general, no pure polymer exists that shows all the required mechanical and barrier characteristics for all packaging applications, so combinations of different layers or blends of polymers are used for particular applications [19,20]. For instance, EVOH film is used in humid environments to provide a barrier against oxygen, although EVOH is a hydrophilic material it serves as a decent barrier against oxygen. To protect EVOH from water, it must be sandwiched in between a material that is hydrophobic and insoluble in water. Hence, multilayers are created with alternating layers of hydrophilic and hydrophobic materials. This strategy can effectively be utilized to reduce the permeation of both, the moisture and oxygen [21,22]. Other than using multilayers, direct blending of polymers is also an important technique to achieving barrier characteristics [23,24,25]. Though these two techniques, polymer blending [24] and multilayer development [20], possess good barrier characteristics and are economical packaging materials, they have high production costs. Moreover, they need unique adhesives for their bonding, which further complicates their formation and are very hard to recycle [26]. Hence, the use of a monolayer coatings is still of great interest in the polymeric industry, along with enhanced barrier and mechanical characteristics [26,27]. Therefore, taking advantages of the transparency and flexibility, polymer matrices can be utilized as a quality barrier by incorporating nanoparticles [14,26,28]. The incorporation of inorganic particles would cause impermeability to permeating gases and thus would offer a tortuous path [10,27,29]. The permeating molecules have to travel a longer distance along the axis of the particle until it finds a polymer or free volume or defect to diffuse to other side; hence permeation would be reduced [29,30,31,32,33]. A reduction in the permeation depends on the concentration and aspect ratio of the nanoparticles within the polymer matrix [30,31,34,35,36].

In this work, poly(vinyl butyral) is selected as the polymer matrix because it is easily processed, offering a high flexibility and excellent adhesion to almost all kinds of materials. Moreover, PVB can maintain high transparency along with good mechanical stability.

Synthetic mica is a fluorine substituted mineral and is found to exist in large and heavy particles due to its high molecular weight [37,38,39]. The large particle size can yield a high aspect ratio, making it useful for barrier applications, and its high molecular weight will help in gaining a preferable horizontal orientation. This powder has very good surface to thickness ratio and can be effectively utilized in reducing gas permeability [40]. Synthetic mica flakes are used as filler due to their decently high aspect ratio. These fillers are incorporated in the soft matrix of PVB and are studied as moisture barrier layers.

## 2. Experimental Section

### 2.1. Materials

PVB (Butvar B-98) was purchased from Aldrich Chemistry,(Darmstadt, Germany) and was used as received. Synthetic mica in the form of flakes (synthetic fluorphlogopite) was obtained from Eckart GmbH, Hartenstein, Germany. Benzyl alcohol was purchased from Thermo Fisher GmbH (Kandel, Germanh). Poly (3-hexylthiophene) (P3HT) was obtained from Merck GmbH, Darmstadt, Germany. PET Melinex ST504 was bought from DuPont Teijin Films U.S. (Discovery Drive Chester, VA, USA) and used as a flexible substrate.

### 2.2. Methods

#### 2.2.1. Preparation of Protective Layer

PVB 30 wt% was dissolved in benzyl alcohol and stirred on hot plate at 80 °C until a clear and homogenous solution was obtained. In the second step, mica flakes were added to the solution with 5, 15, 25 and 35 vol% concentrations and stirred using a planetary mixer for few hours. PET films were used as a substrate and coating was performed using a doctor blade (ZAA 2300—manufactured by Zehntner Testing Instruments, Switzerland). Just before coating, the solution was exposed to a vacuum in order to eliminate entrapped gas bubbles during coating. The temperature on the coating surface was maintained at 30 °C. After coating, the wet layers were allowed to remain on the coating surface for few minutes, so that the filler set and adopt a preferred orientation due to their high density. Finally, the layers were positioned in an oven overnight at a temperature of 80 °C for drying. After drying, films were peeled-off from the PET substrate and used for barrier characterization as free-standing films. For permeability measurements, films with a thickness of around ~70–100 µm were used.

#### 2.2.2. Preparation of P3HT Films for Degradation Test

A 5 vol% solution of P3HT was prepared in o-xylene and a thin film was prepared on a PET substrate using a doctor blade. The prepared film was annealed at 140 °C on a hot plate under ambient conditions.

#### 2.2.3. Encapsulation of Organic Photovoltaic Devices

An inverse structure of a bulk heterojunction organic solar cell (OSC) was prepared on a glass substrate with a layer sequence of glass/ITO/ZnO/P3HT:PCBM/PEDOT:PSS/Ag. Prepared OSC devices were encapsulated with PVB/glass flakes-based barrier using a UV-cured adhesive. ITO coated glass was used as a substrate material which had a sheet resistance of 21 Ω/sq. The top silver electrode was deposited using a thermal evaporation method. The rest of the layers were coated in solutions under ambient conditions using the doctor blading method. The layer of ZnO (N10, purchased from Avantama, Stäfa, Switzerland) was annealed at 120 °C in air for 5 min. The PCBM:P3HT was mixed at a ratio of 0.8:1 (wt/wt) and dissolved in o-xylene:1-methylnaphthalene (19:1, *v*/*v*) solution. A coating of poly (3,4-ethylenedioxythiophene):poly(styrene sulfonate) (PEDOT:PSS) was formed using HTL Solar 388 diluted in water at a ratio of 1:1. Finally, the deposition of silver (Ag) (100 nm) as the top electrode was carried out via thermal evaporation in an ultrahigh vacuum (10^−4^ Pa) through a mask to define an OSC active area of 0.1 cm^2^. Initial current (J) and voltage (V) characteristics, as well as power conversion efficiencies, were measured in a nitrogen atmosphere. After that, the OSC device was encapsulated by lamination with PVB/glass flakes-based barriers using commercial epoxy-based adhesive (DELO Katiobond LP655) that was subsequently cured with UVA light (~400 nm) for 2 min.

### 2.3. Characterization

#### 2.3.1. Microscopic Analysis

Microscopic analyses were carried out through an MX51 optical microscope (Olympus Japan) to determine the size distribution of the flakes.

#### 2.3.2. Spectroscopic Analysis

The transparency of the films in the visible region were investigated using a Shimadzu UV-1800 spectrophotometer equipped with an integrating sphere. Infrared spectra were recorded in transmission mode with a Fourier transform infrared (FTIR) spectrophotometer (Bruker ALPHA-P) operating with OPUS 7.2 software (Bruker, Ettlingen, Germany). Spectra were obtained using 128 scan summations at a 4-cm^−1^ resolution.

#### 2.3.3. Permeability Measurements

Permeability of moisture was measured using an M7002 water vapor permeation analyzer (SYSTECH Illinois, Thame Oxfordshire, UK), which can detect up to 0.02 g/m^2^⋅day or 0.002 g/m^2^⋅day, depending on the sample size. Permeability of oxygen was measured through an oxygen permeation chamber equipped with an optical oxygen sensing spot, PSt9 (Manufactured by PreSens Precision Sensing GmbH, Regensburg, Germany) with a detection limit of 0.1 cm^3^/m^2^⋅day⋅bar.

#### 2.3.4. Bending of the Barrier Layers

A dynamic cyclic bend testing machine was used for fatigue testing, and was developed in-house. In this test, one side of the barrier film was fixed and the other was moving linearly back and forth, thus producing a cyclic film between its adjustable minimum bending radius and planar shape. For this flexible test, the sample size was 3 × 10 cm^2^, and for each test, three samples were utilized. After the bending test, WVTR was carried out on samples with a size of 3 × 3 cm^2^, which were cut from the middle of the bent film.

#### 2.3.5. SEM Images

A JSM-7610F from JEOL with a secondary electron image detector was used for recording scanning electron microscopy (SEM) images. SEM images were taken at an accelerating voltage of 2 kV, with a working distance of 6 mm in the low probe current mode (~65 nA). For SEM cross-sectional images, the samples were broken at a low temperature, i.e., in liquid nitrogen.

#### 2.3.6. Accelerated Lifetime Experiments

The life of the OSC devices was determined in a controlled environment by accelerated lifetime sun tests, where the effects of the barrier layers were observed. The devices were further encapsulated with an optimized PVB/mica composite layer and a commercial barrier (Mitsubishi barrier film with a thickness of 70 µm and a definite WVTR of 3 × 10^−3^ g⋅m^−2^⋅day^−1^) to compare the data of performance. A lamination process was carried out to encapsulate the barrier films on top of the devices using a UV-cured adhesive (DELO Katiobond LP655, DELO, Munich, Germany) in the glove box.

To examine light degradation, the encapsulated devices were positioned in the continuous irradiation chamber of a SUNTEST XXL+ sun simulator (Atlas Materials Testing Technology GmbH, Linsengericht, Germany) with a daylight filter under ambient air. The source of light was a Xenon lamp with an irradiation intensity of 1000 W/m^2^ in the range of 300–400 nm. The temperature of the chamber was kept at 65 °C.

During the ageing experiments, the LOT solar simulator (Class AAA) was used to measure the characteristics of current–voltage and power conversion efficiencies of the solar cells at 1000 W/m^2^. For this particular purpose, the solar cells were taken out of the respective ageing chambers and put back after the measurements.

## 3. Results and Discussion

### 3.1. Optimization of PVB/Mica Flakes Layers

#### 3.1.1. Analysis of PVB/Mica Coating

**Figure 1:**
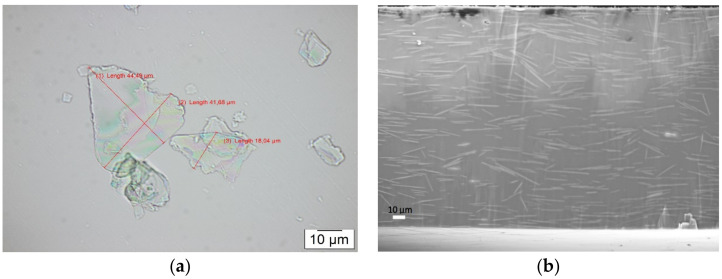
(**a**) Optical micrographs of mica powders, showing size of the flakes, (**b**) SEM cross section of PVB filled with 25 vol% of mica flakes showing random orientation of the flakes.

Flakes of mica were analyzed under a light microscope to confirm the size and shape distribution. Figure 1a shows a microscopic image of the flakes, where the size of flakes is in micrometer range, with an average length of ~40 µm and the thickness is found to be around 1 µm. This implies an aspect ratio of ~40 and, with this type of shape and size, the powder is ideally suited for reducing gas permeability by creating longer paths for diffusing molecules. Figure 1b shows an SEM cross section of PVB filled with flakes with a concentration of 25 wt%. It is observed that flakes within the PVB matrix have an almost uniform distribution with irregular orientations.

#### 3.1.2. FTIR Analysis of Prepared Composite

**Figure 2:**
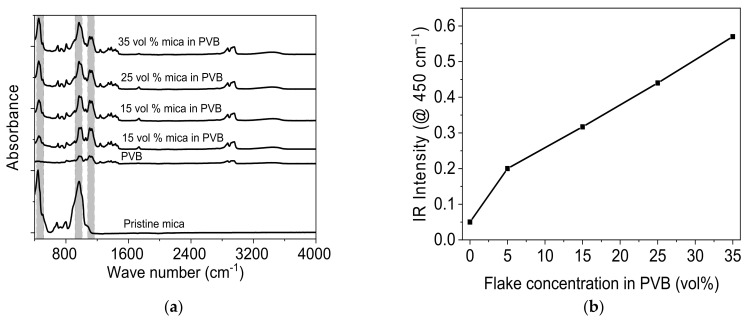
FTIR spectra of PVB filled with mica at different concentrations. (**a**) Spectra of mica powder and layers pristine PVB and PVB filled with 5–35 vol% mica. (**b**) IR peak intensity at 450 cm^−1^ wave number, which shows linear increase with increasing mica concentration (5–35 vol%) in PVB.

FTIR spectra of the layers of PVB filled with mica were recorded for each coating after the complete evaporation of solvent. Pure PVB shows characteristic peaks at around 1000 cm^−1^ and 1200 cm^−1^, as shown in Figure 2a. These peaks are attributed to C-O stretching and H-C-H bending [41,42], whereas mica powder shows characteristic peaks near 450 cm^−1^ and 970 cm^−1^, these characteristic peaks refer to silicate layers, particularly referring to Si-O bending and stretching bonds [4]. The mica powder with vol% of 5–35 were added to PVB. FTIR spectra confirmed the addition of mica powder into the PVB, as a gradual increase in characteristic peaks near to 450 cm^−1^. This continued increasing by increasing the powder content of the PVB matrix.

#### 3.1.3. Barrier Characteristics of Layers

**Figure 3:**
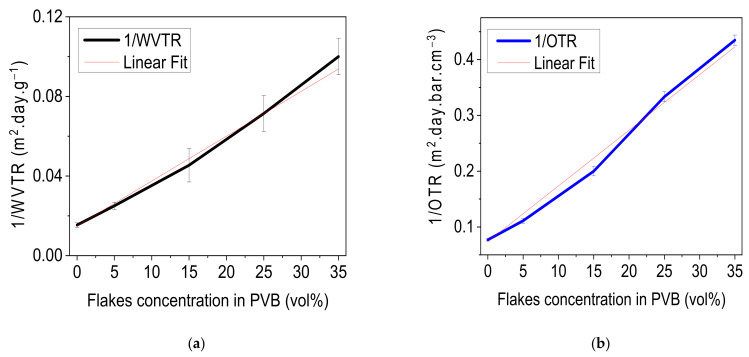
Barrier characteristics of PVB/mica composite, (**a**) inverse of WVTR (black curve), and (**b**) inverse of OTR (blue curve). Both curves show linear barrier improvement as a function of flake concentration.

Permeation curves confirm that pristine PVB is not a good barrier against moisture and exhibits a WVTR of 65 g m^−2^ day^−1^ and the systematic increase in flake concentration generates an extensive path and moisture molecules take longer for diffusion; thus, permeation of moisture decreased [43]. Layers of PVB with flake concentrations of 5, 15, 25 and 35 vol% exhibited moisture permeation of 40 g m^−2^ day^−1^, 22 g m^−2^day^−1^, 14 g m^−2^ day^−1^, and 10 g m^−2^ day^−1^, respectively. This results in an 80% reduction in moisture permeation as compared to pristine PVB layer. Similarly, for oxygen permeation, PVB layers filled with 5, 15, 25, and 35 vol% showed oxygen permeation of 9, 5, 3, and 2.3 cm^3^⋅m^−2^⋅day^−1^⋅bar^−1^, respectively, whereas pristine PVB has an oxygen transmission rate of 13 cm^3^⋅m^−2^⋅day^−1^⋅bar^−1^. In both cases, reduction in permeation increased linearly with increasing flake concentration in the layer (Figure 3).

#### 3.1.4. Validation of Barrier Characteristics

The current experimental data were further compared with the theoretical permeation model proposed by Bharadwaj et al. [44]. A comparison of experimental data with theoretically calculated values was performed. The calculation of tortuosity factor considering volume fraction of the particles, layer thickness, and aspect ratio, along with order parameter based on the orientation of particles within the polymer matrix, were all calculated from Bharadwaj’s model. As the size distribution of the mica flakes was large (as shown in Figure 1a), ranging between 20 µm and 50 µm in lateral length, the large size distribution means that the exact aspect ratio might be between 20 and 50; thus, the calculations of Bharadwaj model were done for this range. The order parameter (S), as suggested by Bharadwaj et al. [44], is an average orientation of the filler and is taken from Figure 1b using Equation (1) and calculated to be *S* = 0.11.

(1) $$S=\frac{1}{2}\left(3cos^{2}\theta -1\right).\;$$

**Figure 4:**
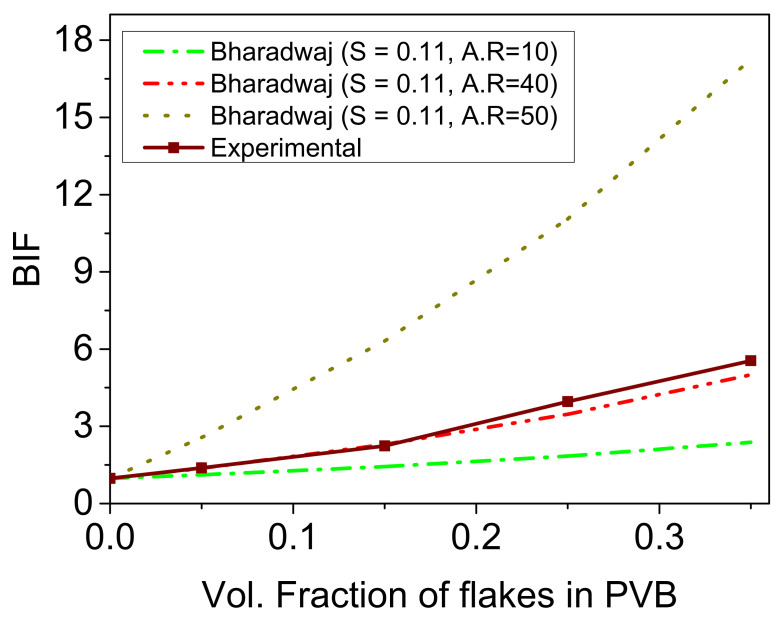
Experimental relative permeability of prepared composites compared with theoretically calculated relative permeability based on Bhardwaj’s model for different aspect ratios (AR) of fillers.

Theoretical and experimental barrier improvement factor (BIF) of the layers is calculated using Equations (2) and (3), respectively.

(2) $$BIF=\frac{Permeability{\;}_{polymer}}{\left(\frac{1-{ø}_{s}}{1+\frac{L}{2W}{ø}_{s}\left(\frac{2}{3}\right)\left(S+\frac{1}{2}\right)\;}\right)}$$

(3) $$BIF=\frac{Permeability{\;}_{polymer}}{Permeability_{\left(composite\right)}}$$

where *ø_s_* represents the volume fraction of the fillers in the composite, *L* and *W* represent the length and width of the fillers (aspect ratio). A comparison of the current experimental values to Bhardwaj’s theoretical model are shown in Figure 4. The experimental values are generated and are in comparison to different aspect ratios, i.e., 10, 40 and 50. The results show that the experimental results are in good accordance with the aspect ratio of 40, which is also in accordance with the average aspect ratio calculated from Figure 1. Therefore, based on this comparison, the barrier quality of the PVB filled with mica fillers with an aspect ratio of 40 was validated.

#### 3.1.5. Transparency of the Layers

**Figure 5:**
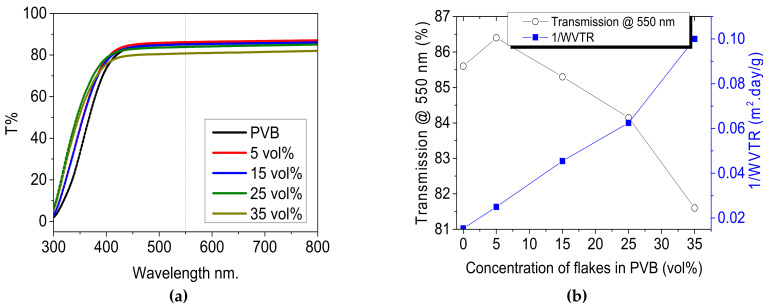
UV-vis spectra of PVB filled with mica at different concentrations. (**a**) Total transmittance spectra of PVB and its composite, (**b**) kinetics of decrease of transmittance @ 550 nm (open circle in black) for PVB filled with 5 vol% to 35 vol% of mica with corresponding 1/WVTR values (closed square in blue).

UV-vis spectra of PVB filled with mica are shown in Figure 5a. Pristine PVB exhibited a transparency of 85% in the visible region. A slight increase in transmittance was observed with the addition of mica fillers, and this increase was attributed to a loss of reflectance in the pristine PVB. Furthermore, the transmittance of the layers decreased linearly with increased filler content. The layers containing 5, 15, 25 and 35 vol% fillers showed transmittances of 86.4, 85.3, 84.15, and 81.6%, respectively. The transmittance dropped to 81% with 35 vol% of filler addition to PVB. This loss of transmittance can be attributed to either layers’ surface roughness or a mismatch of the refractive indices of the polymer and fillers, or a scattering of light due to micronized fillers within the layers. The latter seems the most of the obvious option as the transmittance decreased linearly with increasing filler content. Figure 5b shows the relationship between transparency and the inverse of the WVTR. The results show that relation between the barrier quality and transmittance was inversely proportional, because as the filler contents increased, the barrier quality increases. The choice must be made for optimum required properties, as just 6% loss of transmittance can lead to a gain of 80% better barrier quality with 35 vol% filler content.

#### 3.1.6. Bendability of the PVB/Mica Layers

**Figure 6:**
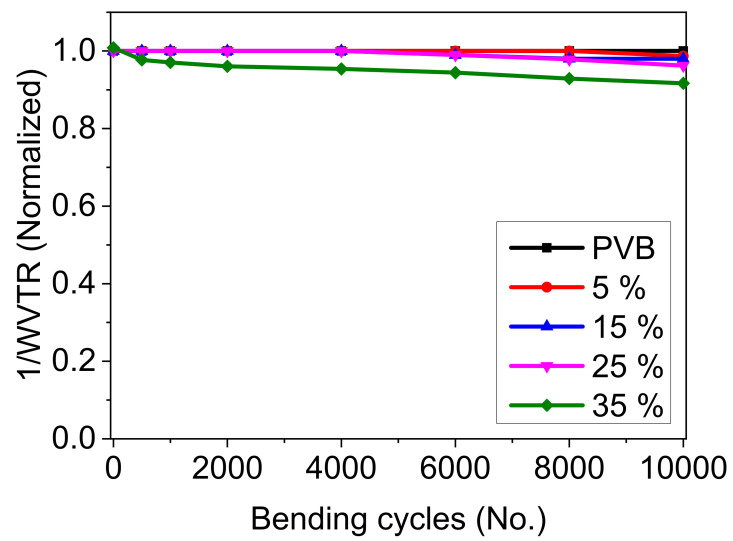
Permeation in terms of normalized 1/WVTR of the PVB layer containing flakes from 5–25 vol% after 10,000 bending cycles at a radius of 3 cm.

The PVB/mica composite layers incorporated with different flake concentrations were subjected to bending cycles to check if the layers were still flexible. All layers were subjected to 10,000 bending cycles at a bending radius of 3 cm. The pristine PVB shows a highly flexible nature and does not lose its barrier properties. This can be attributed to the highly flexible nature of the matrix, as the Young’s modulus of PVB is E(PVB) = 2.36 MPa [4]. The normalized WVTR values of the layers subjected to bending cycles are shown in Figure 6. The results suggest that the layers with 5 vol% maintain their initial permeation values and show no degradation, whereas layers with 15 vol% and 25 vol% slightly degrade and lose <5% of their initial barrier properties. The layer with 35 vol% exhibits around 10% degradation in terms of initial performance. Based on these observations, we conclude that PVB shows a very good adhesion with mica flakes and this adhesion keeps flakes intact in the matrix while bending as the flakes exert tensile and compressive forces simultaneously. Due to the bending of layers of up to 25 vol% of flakes, no deterioration was observed. Increasing flake concentrations above that, on one hand, reduces permeability but may induce brittleness leading to a faster degradation of the layer. One of the possible reasons for the faster degradation may be generation of micro-sized defects while bending. The other possible reason could be a loss of adhesion between the flakes and the matrix at higher loading concentrations, which ultimately lead to degradation. These reasons are both based on assumptions and no experimental work has been carried out.

### 3.2. Encapsulation of P3HT Films

**Figure 7:**
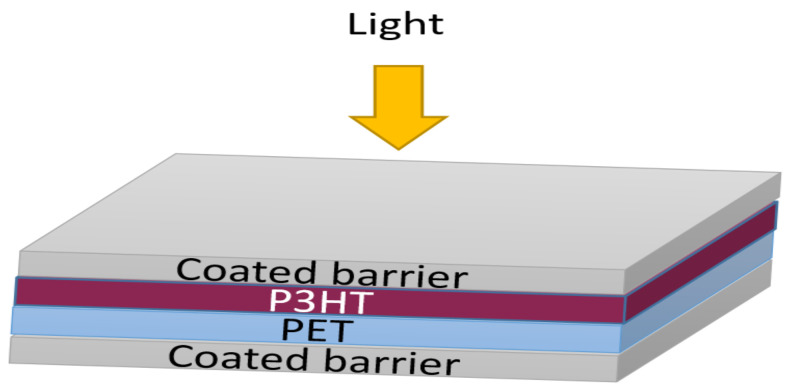
Schematic diagram P3HT film deposited on PET (125 µm) substrate coated from both sides with barrier layers, where coated barrier represents PVB and PVB/mica composite.

In the next stage, optimized layers were used for analyzing the stability of P3HT films. P3HT was used because of its wide applications in the OPV industry as it is a well-characterized material [45]. For this reason, P3HT was coated on a PET substrate and subsequently coated from both sides by pristine PVB and PVB/mica composite (25 vol%) barrier layers, as illustrated schematically in Figure 7.

**Figure 8:**
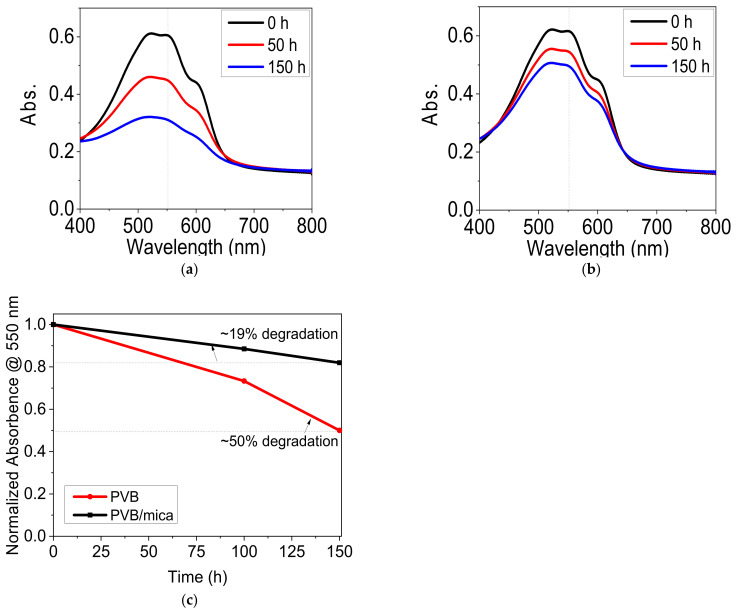
UV-vis spectra of directly coated P3HT films on PET substrate from both sides with (**a**) a pristine PVB (100 µm thick) and (**b**) PVB filled with 25 vol% of mica flakes (~100 µm thick) and (**c**) normalized absorbance loss at 550 nm of P3HT coated with pristine PVB and PVB filled with flakes for an illumination time of 150 h with sun simulator light in ambient air.

The samples were positioned in a sun simulator chamber and constantly illuminated with light in ambient air. UV-vis spectra at different time intervals were used to analyze the rate of photo-oxidation as well as the absorbance loss of the P3HT film at a wavelength of 550 nm. Figure 8a,b shows the absorbance of P3HT film coated with pristine PVB and PVB filled with flakes, respectively. Figure 8c shows the absorbance loss of P3HT at a wavelength of 550 nm. Fifty-percent absorbance loss was observed within 48 h of illumination for the P3HT film coated with pristine PVB. This was because pristine PVB showed a high oxygen permeation, which resulted in the degradation of P3HT under illumination. On the other hand, P3HT film encapsulated with the composite of PVB and mica showed a loss of 20% within the same exposure time, which corresponded to an increase of 60% in P3HT stability against photobleaching. These results confirm that P3HT shows better stability under light illumination when directly coated with PVB/mica composite. It can be extracted from the results that the coating of PVB and mica can protect organic solar cell devices for a time period of over 100 h under ambient conditions, which is sufficient for a short-term protection, e.g., time between production of solar cells and their transportation for final laminations into glass windows, etc. [9].

### 3.3. Encapsulation Organic Solar Cells

**Figure 9:**
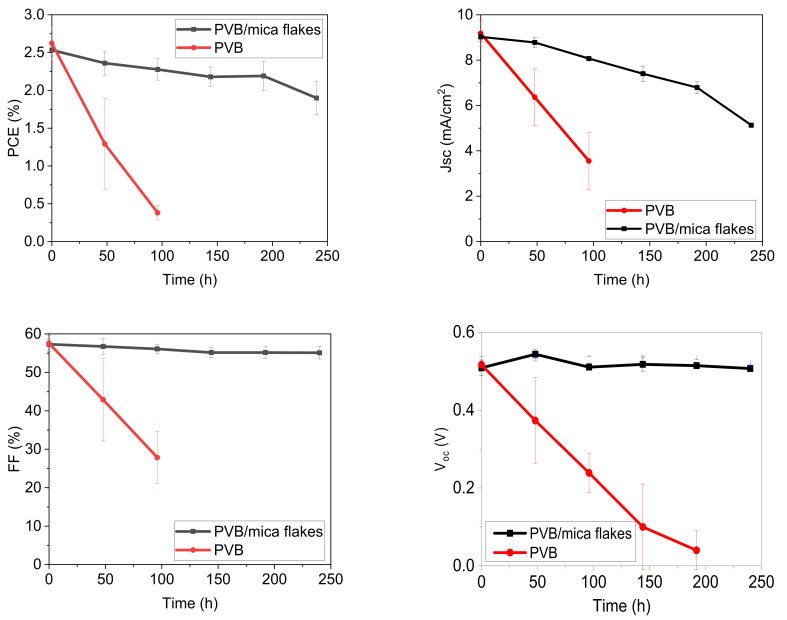
Sun degradation test of P3HT:PCBM-based solar cells encapsulated in barrier films, each having thickness of 150 µm: pristine PVB film and PVB film filled with 25 vol% of mica flakes (AR = 40). Jsc: short circuit current. Voc: open circuit current. FF: fill factor. PCE: power conversion efficiency.

A sun simulator (1000 W m^−^^2^ @ 65 °C temperature) was used to determine the sun degradation test. For this purpose, OSC devices were encapsulated with pristine PVB and PVB filled with mica flakes. The samples were subjected to constant irradiation. The key photovoltaic parameters (J_sc_, FF, V_oc_ and PCE) are shown in Figure 9 and the exact values are mentioned in the Supplementary Materials (Table S1). The results show that the encapsulated device with pristine PVB film rapidly degraded in terms of both J_sc_ and FF, and hence the PCE of the device degraded and reached 80% of its initial performance within less than 20 h of testing. This result clearly indicated a poor barrier performance of pristine PVB films; the OSC device failed. The device encapsulated with PVB/mica flake composite coating showed a degradation of only 20% in PCE after 240 h of irradiation. This loss in PCE was due to the loss of J_sc_, while V_oc_ and the fill factor remained stable. This suggests that degradation was mainly caused by the diffusion of oxygen through the barrier coating, which was the main reason for the subsequent photo-induced reaction with the active layer [2,43]. This can also be observed in jV curves as shown in the Supplementary Materials (Figure S1). Further, this can be noted by the fact that the oxygen amount that diffuses through the barrier within 240 h at 65 °C is adequate to photo-oxidize about 1% of the thiophene rings in the active layer, which is the damage at which P3HT:PCBM cells have been reported to lose around 20% of their initial performance [46].

## 4. Conclusions

A coatable barrier layer prepared from a solution of PVB incorporated with micro-sized mica powder was developed and demonstrated as a potential encapsulation for enhancing the stability of organic solar cells against photo bleaching. The layers of PVB/mica remained highly transparent in the visible region. A linear increase in barrier quality was observed when the concentration of flakes was increased. A barrier improvement factor of ~7 was achieved against both oxygen and moisture. The layers exhibited an ultra-flexible nature and did not show any significant deterioration in barrier quality after 10,000 bending cycles. With encapsulation, an increase of 60% in the stability of P3HT against photo bleaching was observed with the PVB/mica barrier layer. A lifetime extension was also observed when OSC devices were encapsulated with such barrier coatings. A lifetime of about 240 h was achieved when OSC devices were encapsulated with PVB/mica flake films, which is around 12 times longer compared to devices encapsulated with pristine PVB. These results suggest that the PVB matrix with mica flakes has the high potential to be used for short-term encapsulation under ambient conditions for thin film organic solar cells.

## Data Availability

Data sharing not applicable.

## Supplementary Materials

The following are available online at https://www.mdpi.com/article/10.3390/ma14102496/s1, Figure S1: J-V curves of the encapsulated organic solar cells under sun degradation test, (a) encapsulated with a PVB film, and (b) with a PVB/mica flake film as constant irradiation degradation at 1 sun and ambient air (right), Table S1: Solar cell parameters before and after sun irradiation test (testing time for PVB/mica flakes is ~240 h).
