# Supplementary material for: Solution Processed PVB/Mica Flake Coatings for the Encapsulation of Organic Solar Cells
Source: Materials (Basel). 2021 May 12;14(10):2496. doi: 10.3390/ma14102496 (PMC8151763; doi:10.3390/ma14102496)
Supplement: Supplementary file 1 [file materials-14-02496-s001.zip › materials-1207467-supplementary.pdf]

materials

MDPI

Supplementary Information

Solution coated PVB/mica flake coatings for the encapsulation of organic solar cells

Iftikhar Ahmed Channa [1,*], Ali Dad Chandio [1], Muhammad Rizwan [1], Aqeel Ahmed Shah [1], Jahanzeb Bhatti [1,2], Abdul Karim Shah [3], Fayaz Hussain [4], Muhammad Ali Shar [5,6] and Abdulaziz AlHazaa [5,7]

[1] Department of Materials and Metallurgical Engineering, NED University of Engineering and Technology, University road Karachi 75270, Pakistan;alidad@neduet.edu.pk (A.D.C.); engr.rizwan@neduet.edu.pk (M.R.);
aqeelshah@neduet.edu.pk (A.A.S.); jahanzebbhatti@neduet.edu.pk (J.B.)
[2] Department of Mechanical Engineering, Faculty of Engineering, University of Malaya, Kuala Lumpur 50603, Malaysia
[3] Department of Chemical Engineering, Dawood University of Engineering and Technology, Karachi 74800, Pakistan; abdulkarim@duet.edu.pk
[4] Modeling Evolutionary Algorithms Simulation and Artificial Intelligence, Faculty of Electrical & Electronics Engineering, Ton Duc Thang University, Ho Chi Minh City 10000, Vietnam; fayaz@tdtu.edu.vn
[5] King Abdullah Institute for Nanotechnology, King Saud University, Riyadh 11451, Saudi Arabia; mashar@ksu.edu.sa
[6] Department of Mechanical & Energy Systems, Faculty of Engineering and Informatics, University of Bradford, BD7 1DP Bradford, United Kingdom
[7] Research Chair for Tribology, Surfaces and Interface Sciences, Department of Physics and Astronomy, College of Science, King Saud University, Riyadh 11451, Saudi Arabia; aalhazaa@ksu.edu.sa
* Correspondence: iftikharc@neduet.edu.pk

Table S1. Solar cell parameters before and after sun irradiation test (testing time for PVB/mica flakes is ~240 h).

	**PVB**		**PVB/mica Flakes**	
	Before irradiation	After irradiation	Before irradiation	After irradiation
PCE (%)	2.64 ± 0.24	0.38 ± 0.1	2.53 ± 0.1	1.9 ± 0.2
J_{sc} (mA/cm^2)	9.17 ± 0.6	3.55 ± 1.3	9.03 ± 0.2	5.3 ± 0.05
FF (%)	57.5 ± 2.1	27.8 ± 6.8	57.3 ± 1.2	55.1 ± 1.6
V_{oc} (V)	0.51 ± 0.01	0.04 ± 0.05	0.51 ± 0.01	0.51 ± 0.01

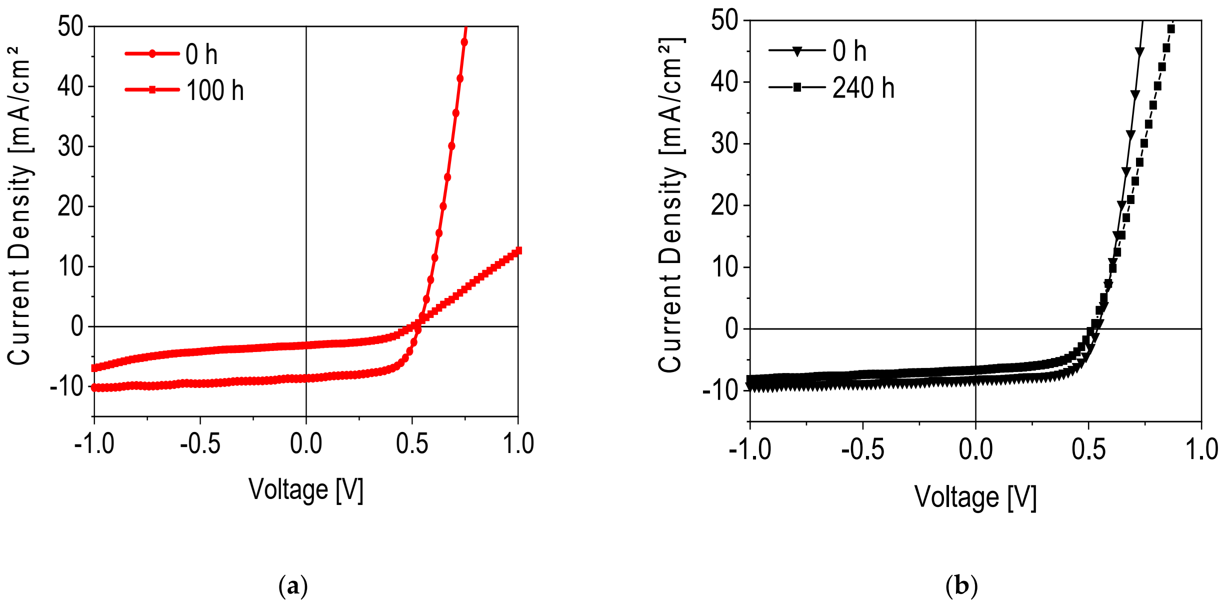

Figure S1. J-V curves of the encapsulated organic solar cells under sun degradation test, (**a**) encapsulated with a PVB film, and (**b**) with a PVB/mica flake film as constant irradiation degradation at 1 sun and ambient air (right).
